# Antibacterial Activity and Mechanism of *Polygonum orientale* L. Essential Oil against *Pectobacterium carotovorum* subsp. *carotovorum*

**DOI:** 10.3390/foods11111585

**Published:** 2022-05-28

**Authors:** Jin Cai, Shiqin Wang, Yichen Gao, Qi Wang

**Affiliations:** 1 Institute of Applied Chemistry, Shanxi University, No. 92 Wucheng Road, Taiyuan 030006, China; 2 Modern Research Center for Traditional Chinese Medicine, Shanxi University, No. 92 Wucheng Road, Taiyuan 030006, China; wangshiqin12247@163.com; 3 The Key Laboratory of Chemical Biology and Molecular Engineering of Ministry of Education, Shanxi University, No. 92 Wucheng Road, Taiyuan 030006, China; 4 School of Life Science, Shanxi University, No. 92 Wucheng Road, Taiyuan 030006, China; gayi136@163.com (Y.G.); wangqi@sxu.edu.cn (Q.W.)

**Keywords:** *Pectobacterium carotovorum* subsp. *carotovorum*, *Polygonum orientale* L., essential oil, process optimization, antibacterial components, antibacterial mechanism

## Abstract

Infected by *Pectobacterium carotovorum* subsp. *carotovorum* (Pcc), the quality of Chinese cabbage could severely decline. Using chemical bactericides to control Pcc could cause food safety problems. Thus, we investigated the optimum extraction conditions, antibacterial activity, chemical compounds and antibacterial mechanism of *Polygonum orientale* L. essential oil (POEO) against Pcc in order to search a new way to control Pcc. The optimum extraction conditions of POEO (soaking time 2.6 h, extraction time 7.7 h and ratio of liquid to solid 10.3 mL/g) were optimized by response surface methodology. The minimum inhibitory concentration (MIC) of POEO against Pcc was 0.625 mg/mL. The control efficiency of protective activity of POEO against Pcc was 74.67~92.67%, and its curative activity was 76.00~93.00%. Then, 29 compounds were obtained by GC-MS; the prime compounds of POEO were phytol, phytone, *n*-pentacosane, 1-octen-3-ol and *β*-ionone. It was verified that, compared with control samples, POEO destroyed cell morphology. It increased surface potential, increased hydrophobicity, damaged cell walls, destroyed the integrity and permeability of cell membrane, reduced membrane potential (MP), and changed membrane protein conformation. It inhibited the activities of pyruvate kinase (PK), succinate dehydrogenase (SDH) and adenosine triphosphatase (ATPase). Briefly, the results of this study demonstrate that POEO showed effective inhibitory activity against Pcc, thus POEO could have potential application in controlling Pcc.

## 1. Introduction

As a typical leafy vegetable, Chinese cabbage is popular with people in China. It has abundant nutrient content, including Ca, Fe, *β*-carotene, kempferol, etc. [1]. *Pectobacterium carotovorum* subsp. *carotovorum* (Pcc) is a gram-negative bacteria, and a phytopathogen that leads to soft rot of Chinese cabbage [2]. During the process of cultivation, transportation and storage, it invades Chinese cabbage through injuries and resides in the vascular tissue, thereby inducing soft rot disease [3]. Infected by Pcc, the leaves and stems of Chinese cabbage become soft and mushy, and then emit an unpleasant smell, which has negative effect on the quality of Chinese cabbage [4].

Traditionally, chemical bactericides such as hypochlorite, formaldehyde solutions, and thiodiazole copper were used to control soft rot [5,6]. However, the long-term application of chemical bactericides can result in pesticide residues, thereby causing harm to human health [7]. As people continue to pay more attention to food safety, it is of great importance to identify a safe and effective method to control Pcc.

Recently, many researchers have concentrated on essential oils (EOs) when searching for natural and safe antibacterial substances to be applied to antibacterial agents for foods. EOs, composed of various secondary metabolites including terpenes, phenols, alcohols, ketones and esters, are extracted from flowers, leaves, seeds, rhizomes, and fruits of aromatic plants [8]. EOs have significant advantages, including low residues, biodegradable natures and low toxicity [7,9]. Because EOs have multiple action targets, they can effectively avoid pathogen proliferation [10]. Several studies have shown that some EOs have significant antimicrobial activities against pathogens, such as *Staphylococcus aureus*, *Listeria monocytogenes* and *Escherichia coli* [11,12,13]. Therefore, EOs serve as a promising natural antibacterial ingredient in developing antibacterial agents for foods.

Recently, *Polygonum orientale* L., a medicinal plant, has generated increased research interest due to its various bioactivities. Hu et al. [14] reported that extract from *P. orientale* had significant insecticidal activity against 13 species of agricultural pests including *Pieris rapae*, *Agrotis ypsilon* and *Mamestra brassicae*. Malik and Barik [15] found that free fatty acids in leaves of *P. orientale* had the potential to be used as a short-term attractant to attract *Galerucella placida*. However, there are no reports related to the antibacterial activity of POEO against Pcc.

Therefore, in this study, the extraction conditions of POEO were first optimized to improve the extraction yield. Secondly, its antibacterial activity against Pcc (in vitro and in vivo) was investigated by measuring its minimum inhibitory concentration (MIC) and the control efficiency. Thirdly, the chemical compounds of POEO were identified. Finally, the antibacterial mechanism was revealed through experiments on cell morphology and cell surface, alkaline phosphatase activity, cell membrane integrity and permeability, membrane potential, membrane protein conformation and key enzyme activities in the respiratory metabolic pathway. These results should provide a basis for potential application of POEO in controlling Pcc.

## 2. Materials and Methods

### 2.1. Materials

**Figure 1:**
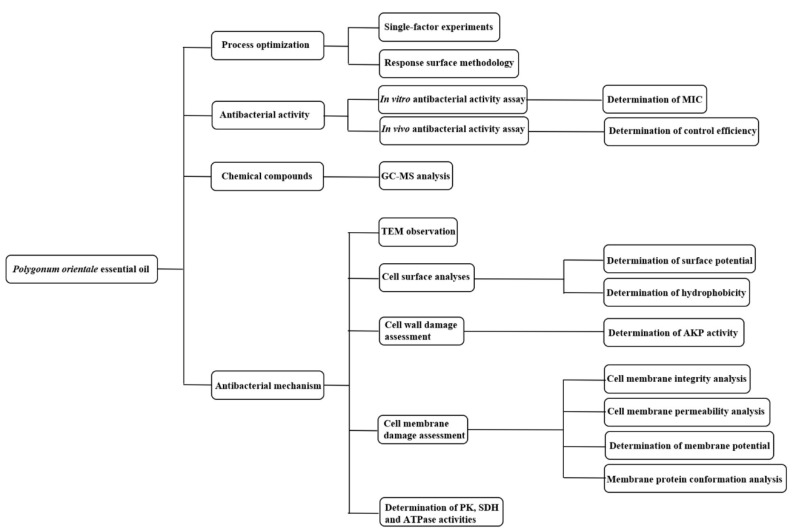
The flowchart of all experiment processes in this study. MIC: minimum inhibitory concentration. GC-MS: gas chromatography–mass spectrometer. TEM: transmission electron microscope. AKP: alkaline phosphatase. PK: pyruvate kinase. SDH: succinate dehydrogenase. ATPase: adenosine triphosphate.

*Pectobacterium carotovorum* subsp. *carotovorum* (CGMCC 1.1000) was obtained from China General Microbiological Culture Collection Center. The strain was incubated by using culture media (5 g/L sodium chloride, 10 g/L peptone, 5 g/L beef extract, 15~20 g/L agar, pH = 7) at 28 °C. In September 2017, *P. orientale* was collected from Taiyuan City of Shanxi Province at 24 °C and 23% relative humidity. Chinese cabbages were purchased from the local market. The seeds were purchased from Dong Fang Zheng Da Seed Co., Ltd., Beijing, China. When all experiments were finished, all experimental materials containing Pcc were sterilized. The flowchart of all experiment processes in this study is presented in Figure 1. The meanings of acronyms appearing in this article are shown in Table S1.

### 2.2. Optimization of the POEO Extraction Process

#### 2.2.1. Single-Factor Experiments

Single-factor experiments were performed to select appropriate levels of factors for the experimental design of the response surface methodology (RSM). The steps of the single-factor experiments were as follows:

##### Soaking Time

To investigate the effect of soaking time on extraction yield of POEO, 100 g crushed *P. orientale* and 800 mL distilled water were added into a flask and immersed for 0, 3, 6, 9 and 12 h respectively. POEO was obtained after 3 h by steam distillation. Through drying, the extraction yield (%) of POEO was calculated by Equation (1).

The yield (%) = the mass of essential oil/the mass of *P. orientale* (1)

##### Extraction Time

To investigate the effect of extraction time, 100 g crushed *P. orientale* was added to 800 mL distilled water and immersed for 3 h, and the POEO was extracted for 3, 6, 9, 12 and 15 h. Through drying, the extraction yield (%) of POEO was calculated by Equation (1).

##### Ratio of Liquid to Solid

To investigate the effect of liquid to solid ratio, 600, 800, 1000, 1200 and 1400 mL distilled water were respectively added into flasks containing 100 g crushed *P. orientale*, and the POEO was obtained at the soaking time with extraction time of 3 h. Through drying, the extraction yield (%) of POEO was calculated by Equation (1).

#### 2.2.2. Response Surface Methodology

Response surface methodology (RSM) with Box-Behnken design (BBD) was used to optimize the extraction process [16]. Based on the results of single-factor experiments (Table 1), BBD was designed and is presented in Table 2. Experimental results were fitted for a second-order polynomial equation: Equation (2).

*Y = β_0_ + β_1_X_11_ + β_2_X_2_ + β_3_X_3_ + β_11_X_1_^2^ + β_22_X_2_^2^ + β_33_X_3_^2^ + β_12_X_1_X_2_ + β_13_X_1_X_3_ + β_23_X_2_X_3_* (2)

In this equation, *Y* is the extraction yield of POEO; *β_0_* is the constant coefficient; *β_1_*, *β_2_* and *β_3_* are the first-order coefficients; *β_11_*, *β_22_* and *β_33_* are the quadratic coefficients and *β_12_*, *β_13_* and *β_23_* are the interaction coefficients. *X_1_*, *X_2_* and *X_3_* are the soaking time, extraction time and ratio of liquid to solid.

The second-order polynomial equation was analyzed by analysis of variance (ANOVA). The 3D response surface plots were obtained by Design-Expert software in order to confirm optimal extraction conditions of POEO.

### 2.3. Antibacterial Activity Assays

#### 2.3.1. In Vitro Antibacterial Activity Assay

In our study, POEO was dissolved in 3% DMSO and diluted by culture media to achieve concentration of 40 mg/mL. Then, 40 mg/mL of POEO solution was prepared with the double dilution method to gain concentrations of 40~0.01 mg/mL in a 96-well plate [17]. Treatment with 3% DMSO was used as a control. The 50 μL Pcc suspension (10^6^ CFU/mL) was added into each well and incubated at 28 °C for 24 h. When the mediums were clean, the minimum concentration suspension was streaked onto plates. When the concentration of essential oil reached MIC, there was no visible Pcc growing on the plate [18].

#### 2.3.2. In Vivo Antibacterial Activity Assay

POEO was dissolved in 3% DMSO and diluted by 1% Tween 20 and sterile distilled water, until the final concentrations of POEO were 20, 10 and 5 mg/mL [19,20]. The stems of Chinese cabbage were cut into pieces of uniform size. The pieces were surface sterilized with 75% ethanol and washed four times with sterile distilled water. Then, a well (4 mm) was made in each piece. Subsequently, antibacterial activity assay was carried out as follows:

##### Protective Assay

For the protective assay, 1 mL of POEO (20, 10 and 5 mg/mL) was uniformly sprayed onto the pieces. After 24 h, 100 μL of Pcc suspension (10^9^ CFU/mL) was inoculated on each well. The control was treated with 1 mL of a mixed solution, containing 3% DMSO and 1% Tween 20. These pieces were cultivated in sterile petri dishes at 28 °C [21]. The degree of rot was measured according to the method presented by Li et al. [22], measuring the size of the rotten area. The control efficiency of protective activity was calculated by Equation (3) on the third day.

Control efficiency (%) = (C − E)/C × 100%(3)

In this formula, C is the degree of rot in the control group and E is the degree of rot in the experimental group.

##### Curative Assay

For the curative assay, 100 μL of Pcc suspension (10^9^ CFU/mL) was inoculated on each well. After 24 h, 1 mL of POEO (20, 10 and 5 mg/mL) was uniformly sprayed onto the pieces. The control was treated with 1 mL of a mixed solution, containing 3% DMSO and 1% Tween 20. These pieces were cultivated in sterile petri dishes at 28 °C [21]. The degree of rot was measured according to the method from Li et al. [22], by measuring the size of the rotten area. The control efficiency of curative activity was calculated by Equation (3) on the third day.

### 2.4. GC-MS Analysis

The chemical compounds of POEO were analyzed by TSQ^TM^ 8000 Evo GC-MS (Thermo Fisher Co., Waltham, MA, USA). The gas chromatograph was equipped with a TG-5MS capillary column (30 m × 0.25 mm × 0.25 μm). The carrier gas was helium with a flow rate of 1.0 mL/min. The inlet temperature was set as 250 °C. The column temperature program was set to rise from 80 °C to 243 °C at 4 °C/min, and kept for 10 min at 243 °C. The sample (1.0 μL) was injected into GC-MS with a split of 10:1, and the range of mass spectra was 10~650 m/z at 70 eV in EI mode. The solvent delay time was 3 min. The standard mixture of *n*-alkanes was injected into the system for calculating retention indices (RIs) by ADMIS software [23]. By comparing RIs with standard values in the NIST Search 2.2 database, the compounds of POEO were identified. The relative percentage of each compound was obtained by GC peak normalization.

### 2.5. Transmission Electron Microscope (TEM) Analysis

The 100 μL of Pcc suspension (10^9^ CFU/mL) was inoculated in a liquid medium (100 mL). Then, POEO was dissolved in 2% DMSO and added into the medium to gain 5 mg/mL POEO solution. Treatment with 2% DMSO only was used as a control. All treatments were incubated for 8 h at 28 °C and 120 rpm. The Pcc cells were collected and washed with physiological saline three times. The cells were fixed in 2% glutaraldehyde for 2 h at 4 °C. After being washed with buffer solution three times, cells were further fixed in OsO_4_ for 2 h and underwent a series of dehydration (5~15 min per stage). Afterwards, the samples were soaked for 2~4 h and embedded in epoxy resin 618. The samples were made into ultrathin sections and double-stained for 15~30 min with uranyl acetate and lead citrate. The changes of morphology were observed by TEM (JEM-1011, JEOL Co., Ltd., Tokyo, Japan) [24].

### 2.6. Cell Surface Analyses

#### 2.6.1. Determination of Surface Potential

The 100 μL of Pcc suspension (10^9^ CFU/mL) was inoculated in a liquid medium (100 mL). Then, POEO was dissolved in 2% DMSO and added into the medium to achieve final concentrations of 1/2MIC, MIC and 2MIC. Treatment with 2% DMSO served as a control. All suspensions were incubated for 8 h at 28 °C (120 rpm). The cells were collected and washed with physiological saline three times. Afterwards, the samples were obtained by resuspending the cells to OD_600nm_ = 0.6. Finally, the zeta potential was determined by a Zetasizer Nano ZS (Malvern Co., Malvern, UK) [25].

#### 2.6.2. Determination of Hydrophobicity

The samples were prepared as described in Section 2.6.1. The 1.5 mL cetane was added respectively into 4 mL samples as experimental groups. The 1.5 mL of physiological saline was added respectively into 4 mL samples as control. After 20 min, the OD values (600 nm) of aqueous phase (3 mL) were measured by UV-visible spectrophotometer (Shanghai Spectrum Instruments, Co., Ltd., Shanghai, China). Equation (4) was used to calculate the hydrophobicity index.

Hydrophobicity index = (1 − OD_1_/OD_0_) × 100% (4)

In this formula, OD_0_ is the OD value of the control group and OD_1_ is the OD value of the experimental group.

### 2.7. Cell Wall Damage Assessment

The effect of POEO on cell walls was investigated by measuring extracellular alkaline phosphatase (AKP) activity [26]. The 100 μL of Pcc suspension (10^9^ CFU/mL) was inoculated in a medium (100 mL). Then, POEO was dissolved in 2% DMSO and added into the medium to gain final concentrations of 1/2MIC, MIC and 2MIC. Treatment with 2% DMSO served as a control. All suspensions were incubated for 8 h at 28 °C and 120 rpm. After centrifuging at 4000 rpm for 10 min, the supernatants were used to measure extracellular AKP activity at 520 nm with an AKP detection kit (Nanjing Jiancheng Bioengineering Institute, Nanjing, China).

### 2.8. Cell Membrane Damage Assessments

#### 2.8.1. Cell Membrane Integrity Analysis

Cell membrane integrity was investigated by a propidium iodide (PI) staining method [27]. The samples were prepared as described in Section 2.6.1. Then, 5 μL of PI and 95 μL of samples were mixed and incubated at room temperature for 30 min in the dark. Afterwards, all groups were washed with physiological saline three times. Finally, the fluorescence intensity was measured using a fluorescence spectrophotometer (F-280, Tianjin Gangdong Technology Development Co., Ltd., Tianjin, China) at wavelengths of 536 nm (excitation) and 539 nm (emission).

#### 2.8.2. Cell Membrane Permeability Analysis

The permeability of cell membrane was investigated by utilizing the *β*-galactosidase method [28] with some modifications. The 0.05 g NaCl, 1.71 g Na_2_HPO_4_·12H_2_O, 0.3 g KH_2_PO_4_ and 0.1 g NH_4_Cl were added into a 100 mL volumetric flask, and sterile distilled water was added to the scale mark. The M9 stock solution was obtained by autoclaving. Then, 0.2 mL MgSO_4_ (1 mol/L), 10 μL CaCl_2_ (1 mol/L), 25 mL lactose solution (20%, *m/v*), 20 mL M9 stock solution and sterile distilled water were mixed to a final volume of 100 mL to obtain the M9 lactose induction medium. The cells of 1mL Pcc suspension (10^9^ CFU/mL) were collected and washed with physiological saline three times. The cells were inoculated in M9 lactose induction medium (10 mL). POEO was dissolved in 2% DMSO and added into the medium to obtain final concentrations of 1/2MIC, MIC and 2MIC. Another group treated with 2% DMSO served as a control. All groups were cultivated for 8 h at 28 °C at 120 rpm, rinsed with *β*-galactosidase reaction buffer (0.8 g NaCl, 0.024 g KH_2_PO_4_, 0.025 g MgSO_4_·7H_2_O, 0.29 g Na_2_HPO_4_·12H_2_O, 0.02 g KCl, 0.39 mL *β*-mercaptoethanol and sterile distilled water; final volume of 100 mL) three times and resuspended to OD_600nm_ = 0.6. After resuspension, 100 μL of 1 mg/mL *o*-nitrophenyl-*β*, *D*-galactopyranoside (ONPG) and 1 mL of Pcc suspensions were mixed and treated in a water bath for 30 min at 28 °C. Finally, the absorbances of supernatants at 405 nm were measured using an UV-Visible spectrophotometer.

#### 2.8.3. Determination of Membrane Potential

The membrane potential (MP) was evaluated using the rhodamine fluorescence method [18]. The samples were prepared as described in Section 2.6.1. Then 5 μL of rhodamine 123 was added to 1.5 mL of sample and the final concentration of rhodamine 123 was 20 μmol/mL. All groups were then incubated (in the dark) for 30 min and washed three times with physiological saline. Finally, fluorescence intensity was measured in a fluorescence spectrophotometer at wavelengths of 505 nm (excitation) and 530 nm (emission).

#### 2.8.4. Membrane Protein Conformation Analysis

The changes of membrane protein conformation were determined by fluorescence spectra assay [29]. The samples were prepared as described in Section 2.6.1. The excitation wavelength was fixed at 280 nm and the slit widths of the excitation and emission raster were 1 nm. By scanning at 323~345 nm, the emission spectra of samples were obtained in a fluorescence spectrophotometer.

### 2.9. Determination of PK, SDH and ATPase Activities

The samples were prepared as described in Section 2.6.1. Then, the samples were treated with an ultrasonic sonifier (JY92-Ⅱ, Ningbo Scientz Biotechnology, Ningbo, China) in an ice bath. The conditions were as follows: power, 200 w; ultrasound interval, 4 s; ultrasound time, 2 s/time; total ultrasound time, 15 min. The samples were centrifugated at 10,000 rpm, and the supernatants were kept at −20 °C. Finally, the PK, SDH and ATPase activities of Pcc exposed to different concentrations of POEO were determined by using a PK detection kit, an SDH detection kit and an ATPase detection kit (Nanjing Jiancheng Bioengineering Institute, Nanjing, China) [30].

### 2.10. Statistical Analysis

Each experiment was performed in triplicate. Duncan’s multiple comparisons test was used to analyze data via SPSS 16.0 software. The results were expressed as mean ± standard deviation.

## 3. Results

### 3.1. Single-Factor Experiments of POEO Extraction

**Figure 2:**
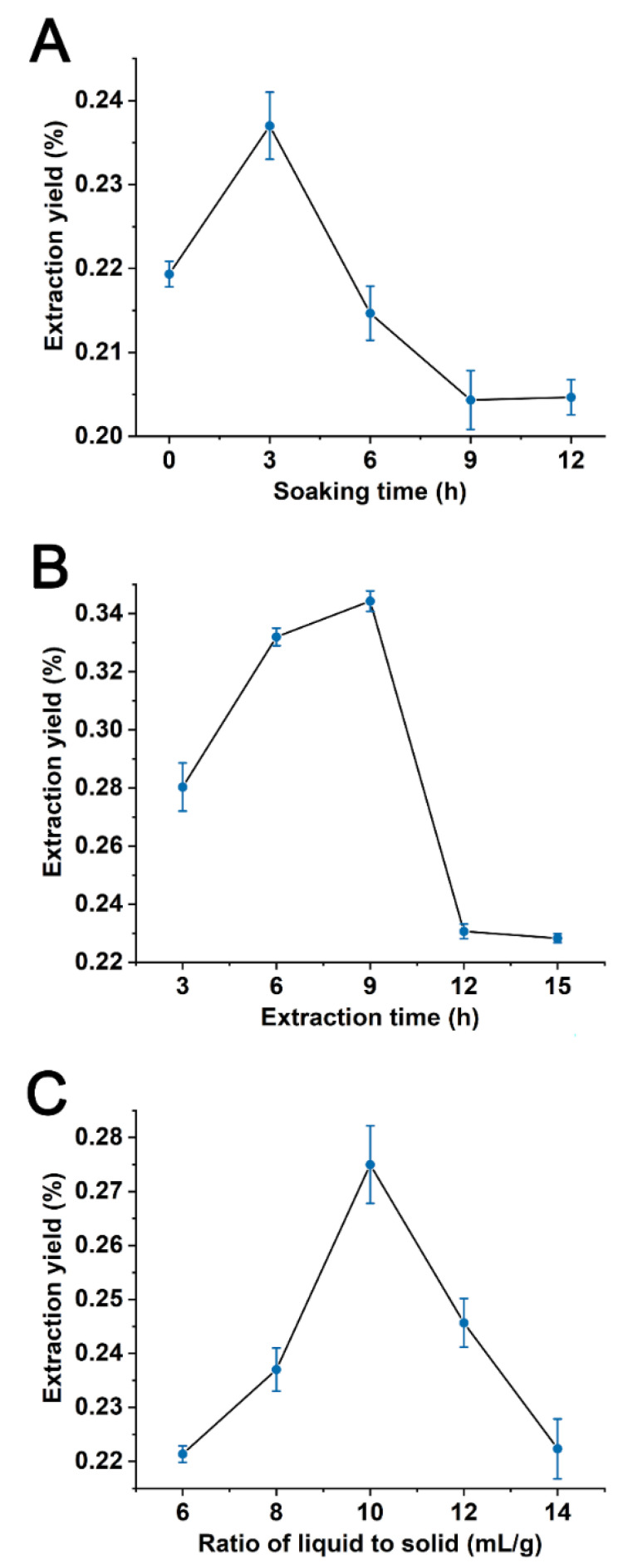
Effects of soaking time (**A**), extraction time (**B**) and liquid to solid ratio (**C**) on extraction yield of *Polygonum orientale* essential oil (%).

The effect of soaking time on extraction yield was studied at 0, 3, 6, 9 and 12 h. In Figure 2A, the extraction yield rose from 0 h to 3 h, reached maximum (0.237%) at 3 h and then significantly decreased. Thus, 0, 3 and 6 h were chosen and served as the three levels of soaking time for BBD.

The effect of extraction time was investigated at 3, 6, 9, 12 and 15 h. In Figure 2B, the yield at extraction time increased from 3 h to 9 h, reached maximum (0.344%) at 9 h and from 9 h to 15 h it decreased. According to these results, 3, 6 and 9 h were selected as the three levels of extraction time for BBD.

The effect of liquid to solid ratio was investigated at 6, 8, 10, 12 and 14 mL/g. In Figure 2C, the extraction yield increased significantly when the liquid to solid ratio rose from 6 to 10 mL/g, reached maximum (0.275%) at 10 mL/g and then declined. Therefore, 8, 10 and 12 mL/g served as the three levels of liquid to solid ratio for BBD.

### 3.2. Optimization of POEO Extraction Conditions

Based on the results of single-factor experiments (Table 1), we designed BBD to gain the optimal extraction conditions for POEO. The 17 experiments are presented in Table 2. The results were subjected to regression analysis, and the second-order polynomial equation was derived (Equation (5)).

*Y* = 0.4072 − 0.0144*X_1_* + 0.0588*X_2_* + 0.0151*X_3_* + 0.00575*X_1_X_2_* + 0.00445*X_1_X_3_* + 0. 00125*X_2_X_3_* − 0.0436*X_1_^2^* − 0. 0509*X_2_^2^* − 0.0601*X_3_^2^* (5)

where *Y* is the extraction yield of POEO. *X_1_*, *X_2_* and *X_3_* respectively represent soaking time, extraction time and ratio of liquid to solid.

The results after analysis of variance (ANOVA) are shown in Table 3. Equation (5) was extremely significant (*p* < 0.0001) and the model was ideal (*F* = 29.86). The lack of fit was not significant (*p* = 0.4349). Correlation coefficient (R-Squared) was found to be 97.46%, indicating low experimental error. Adjusted correlation coefficient (Adj R-Squared) was 94.20%, indicating that the model was able to account for 94.20% of the variation in the response. In addition, the linear term (*X_2_*) was extremely significant (*p* < 0.01) and the linear terms (*X_1_*, *X_3_*) showed significant effects (*p* < 0.05). The quadratic terms (*X_1_^2^*, *X_2_^2^* and *X_3_^2^*) were extremely significant (*p* < 0.01), but the interaction terms (*X_1_X_2_*, *X_1_X_3_* and *X_2_X_3_*) had no significant influence on the extraction yield. The order of impact of each factor was extraction time, followed by ratio of liquid to solid, and then soaking time.

In Equation (5), the coefficients of linear terms (*X_2_*, *X_3_*) and interaction terms (*X_1_X_2_*, *X_1_X_3_* and *X_2_X_3_*) were positive, indicating that they had positive effects on increasing the extraction yield. However, the linear term (*X_1_*) and quadratic terms (*X_1_^2^*, *X_2_^2^* and *X_3_^2^*) had negative effects on the extraction yield of POEO.

**Figure 3:**
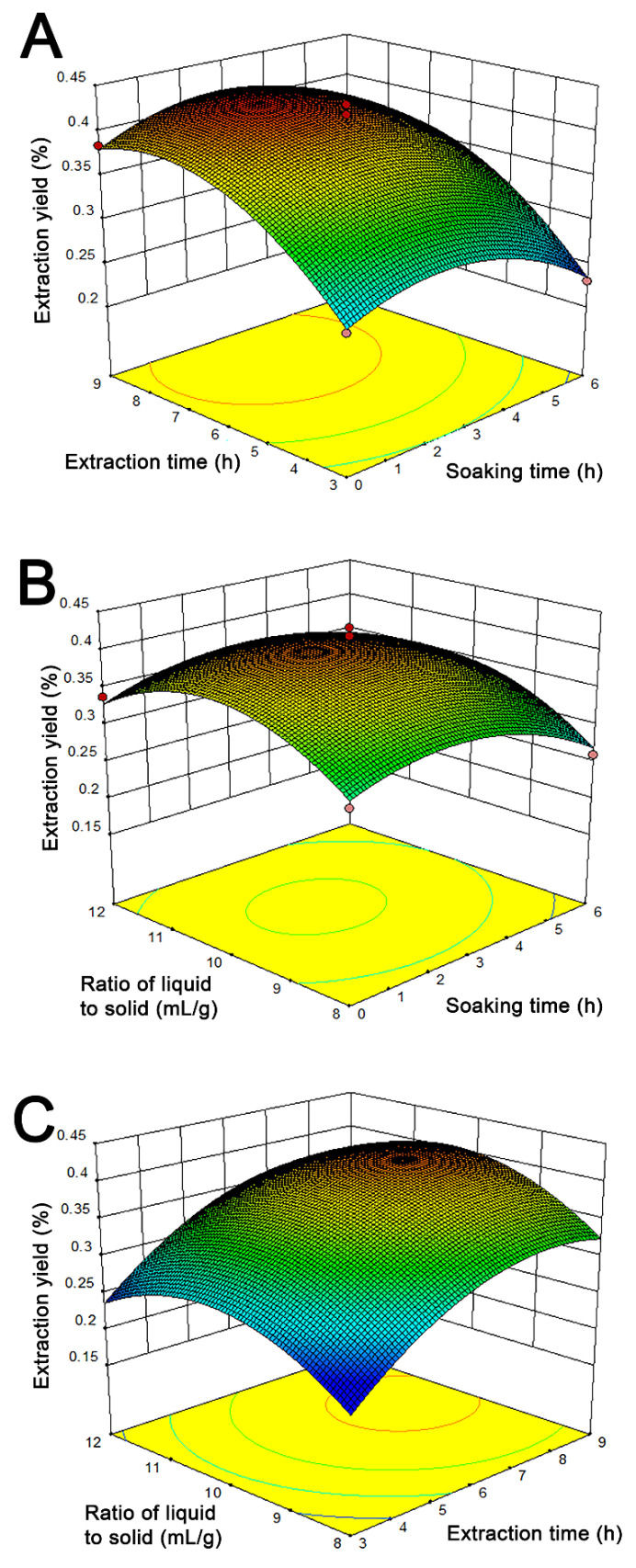
3D response surface plots of the interaction effects between extraction time and soaking time (**A**), liquid to solid ratio and soaking time (**B**), liquid to solid ratio and extraction time (**C**) on the extraction yield of *Polygonum orientale* essential oil.

The 3D response surface plots graphically explore the relative effects of soaking time, extraction time and liquid to solid ratio, as shown in Figure 3. The interaction effect of soaking time and extraction time on extraction yield of POEO is illustrated in Figure 3A. The effect of extraction time on extraction yield was significantly greater than that of soaking time. Figure 3B shows the 3D response surface plot for the influence on extraction yield of soaking time and liquid to solid ratio. The liquid to solid ratio had a greater influence than soaking time on extraction yield. Figure 3C illustrates the combined effect of extraction time and liquid to solid ratio. The effect of extraction time on extraction was stronger than effect of liquid to solid ratio.

Finally, the optimal extraction conditions were 2.64 h soaking time, 7.71 h extraction time and 10.25 mL/g liquid to solid ratio. In order to apply to actual operations, the conditions were revised to 2.6 h soaking time, 7.7 h extraction time and 10.3 mL/g liquid to solid ratio.

### 3.3. Antibacterial Activity of POEO

#### 3.3.1. in vitro Antibacterial Effects of POEO on Pcc

The MIC of POEO against Pcc is shown in Table 4. POEO showed strong antibacterial activity against Pcc at concentrations of 40~0.625 mg/mL, and these mediums were clean. With the decrease of concentration (0.313~0.08 mg/mL), the mediums were gradually turbid because of the growth of Pcc. Treated with 0.04~0.01 mg/mL of POEO, the mediums were completely turbid. In the negative control (3% DMSO), the mediums were also completely turbid. The 0.625 mg/mL suspension was streaked onto plates and there was no growth of Pcc cells after incubation. Thus, the MIC of POEO against Pcc was 0.625 mg/mL.

#### 3.3.2. In Vivo Antibacterial Effects of POEO on Pcc

**Figure 4:**
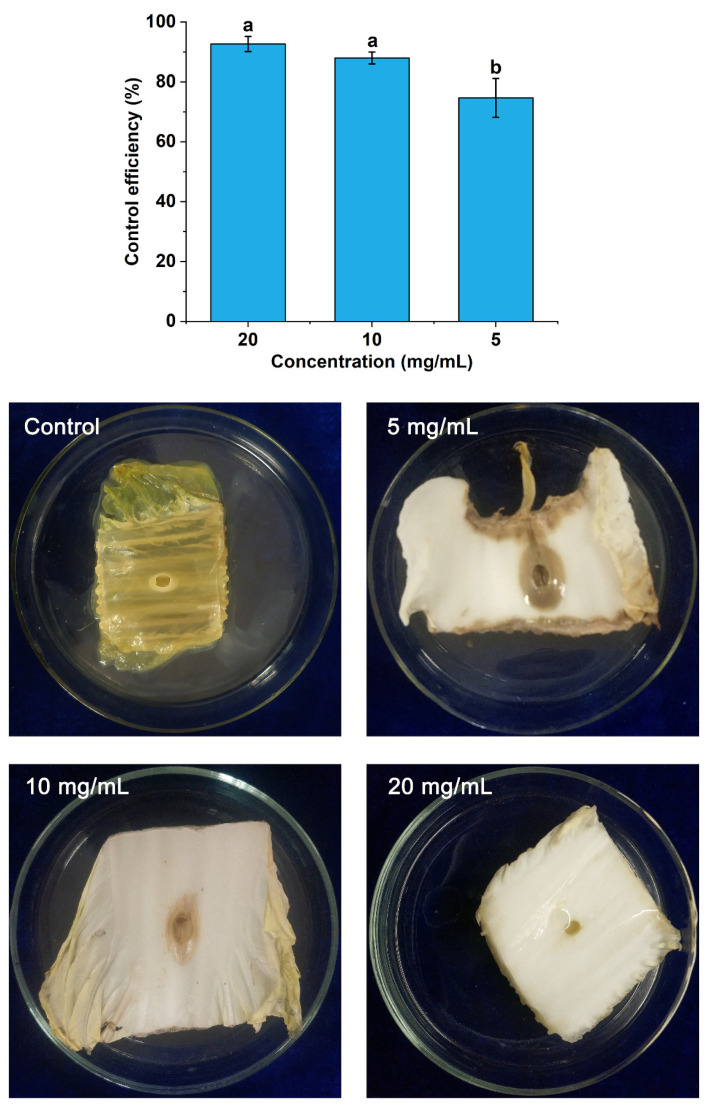
Protective activity of *Polygonum orientale* essential oil against *Pectobacterium carotovorum* subsp. *carotovorum*. The control was treated with 3% DMSO and 1% Tween 20. A significant difference was expressed (*p* < 0.05).

In the protective and curative activity assays, the degree of rot was measured according to the method reported by Li et al. [22] with some modifications. In this study, the size of the rotten area was used to represent the degree of rot and was measured using a ruler. In comparison to the control, a series of POEO concentrations significantly reduced the rot caused by Pcc. As seen in Figure 4, the whole Chinese cabbage stem was completely rotten in the control and the areas of rot were significantly greater than the three experimental groups (5 mg/mL, 10 mg/mL and 20 mg/mL). With the increase of concentrations of POEO, the area of rot was markedly reduced. In the 20 mg/mL group especially, the Chinese cabbage stems were scarcely decayed. The control efficiency of protective activity in the 5 mg/mL group was 74.67%. Compared with the 5 mg/mL group, the control efficiency of protective activity increased to 88.00% and 92.67% after treatment with POEO at 10 mg/mL and 20 mg/mL.

**Figure 5:**
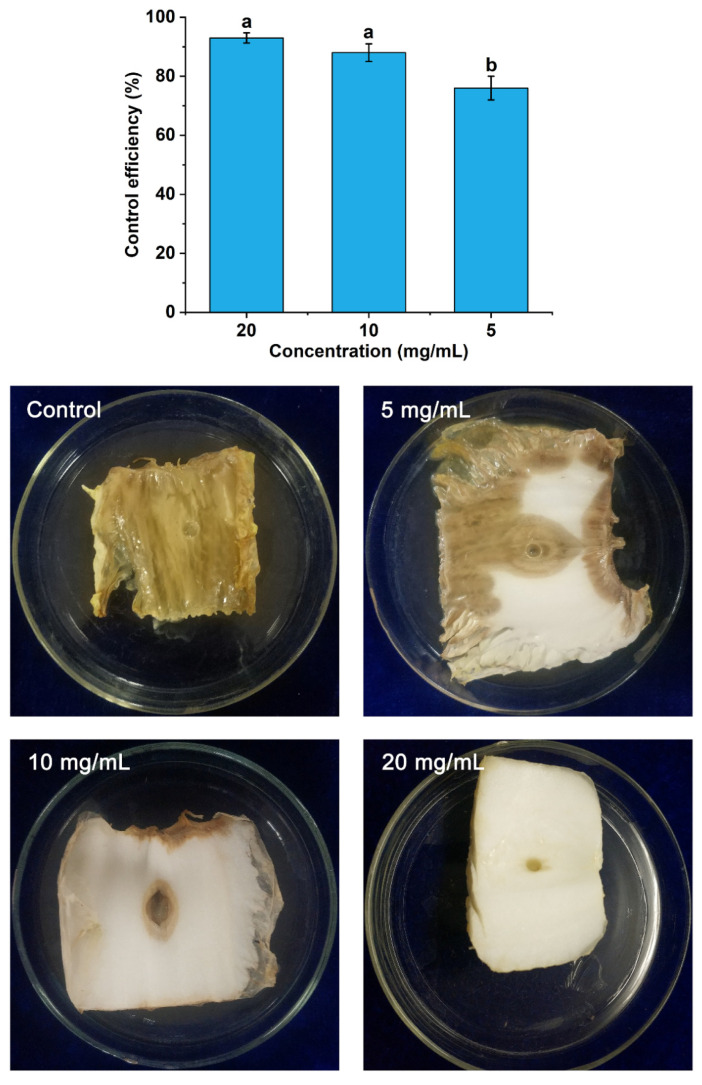
Curative activity of *Polygonum orientale* essential oil against *Pectobacterium carotovorum* subsp. *carotovorum*. The control was treated with 3% DMSO and 1% Tween 20. A significant difference was expressed (*p* < 0.05).

In Figure 5, the Chinese cabbage stem in the control entirely decayed, with a tan color. With the increase of POEO concentrations, the degree of rot was gradually reduced. In the 20 mg/mL group, symptoms caused by Pcc were scarcely observed. The control efficiency of curative activity (5 mg/mL) was 76.00%. Treated with 10 mg/mL and 20 mg/mL, the control efficiency increased to 88.00% and 93.00% respectively.

### 3.4. Chemical Compounds of POEO

Through GC-MS analysis, chemical compounds of POEO were listed in Table 5. We obtained 29 different compounds, and identified 23 compounds that accounted for 94.34% of the total content. The principal compounds in POEO were phytol (23.87%), phytone (17.24%), *n*-pentacosane (12.62%), 1-octen-3-ol (6.75%) and *β*-ionone (6.66%). The 23 identified compounds could be classified into terpenoids (phytol, *β*-ionone, farnesyl acetone, geranyl acetone, isophytol, *β*-cyclocitral, safranal and *β*-homocyclocitral), alkanes (*n*-pentacosane, *n*-heneicosane, *n*-heptacosane, *n*-tricosane, *n*-hexacosane, *n*-heptadecane, *n*-tetracosane and *n*-octadecane), ketones (phytone, 3,5-octadien-2-one and 2-methyl-2-decen-4-one), alcohol (1-octen-3-ol), carboxylic acids (palmitic acid and linoleic acid) and ester (di-isobutyl phthalate). The major classes were terpenoids (40.33%), alkanes (22.58%) and ketones (18.09%).

### 3.5. Effect of POEO on Microscopic Morphology

**Figure 6:**
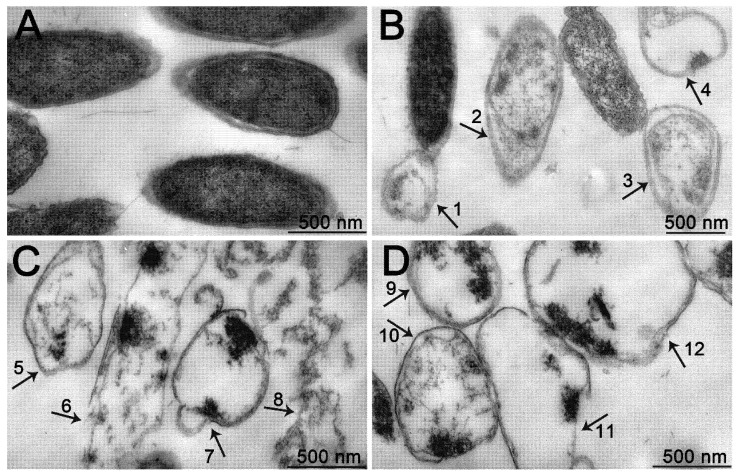
Transmission electron micrographs of *Pectobacterium carotovorum* subsp. *carotovorum* (Pcc) treated with 2% DMSO (control; (**A**): 80,000 ×) and 5 mg/mL of *Polygonum orientale* essential oil ((**B**): 60,000 ×, (**C**): 80,000 ×, (**D**): 80,000 ×). Pcc cells displayed vacuoles (1, 4, 5, 9, 10, 11 and 12), and some showed plasmolysis (2 and 3). Some Pcc cells formed long strips (6) and cyclic shapes (7). Plentiful cell fragments appeared in some Pcc cells (8).

As shown in Figure 6A, the cells in the control group had integral structure and smooth cell membranes, and were plump. Under the action of POEO, the morphology of Pcc changed obviously (Figure 6B–D). Some cells appeared to exhibit the phenomena of plasmolysis (Figure 6B, numbers 2,3). Vacuoles formed due to the leakage of cellular contents (Figure 6B, numbers 1,4; Figure 6C, number 5; Figure 6D, numbers 9,10,11,12). Meanwhile, some cells twisted into different shapes, such as long strips (Figure 6C, number 6) and cyclic shapes (Figure 6C, number 7). Furthermore, some bacteria were completely destroyed, causing the appearance of plentiful cell fragments (Figure 6C, number 8).

### 3.6. Effects of POEO on Cell Surface

**Figure 7:**
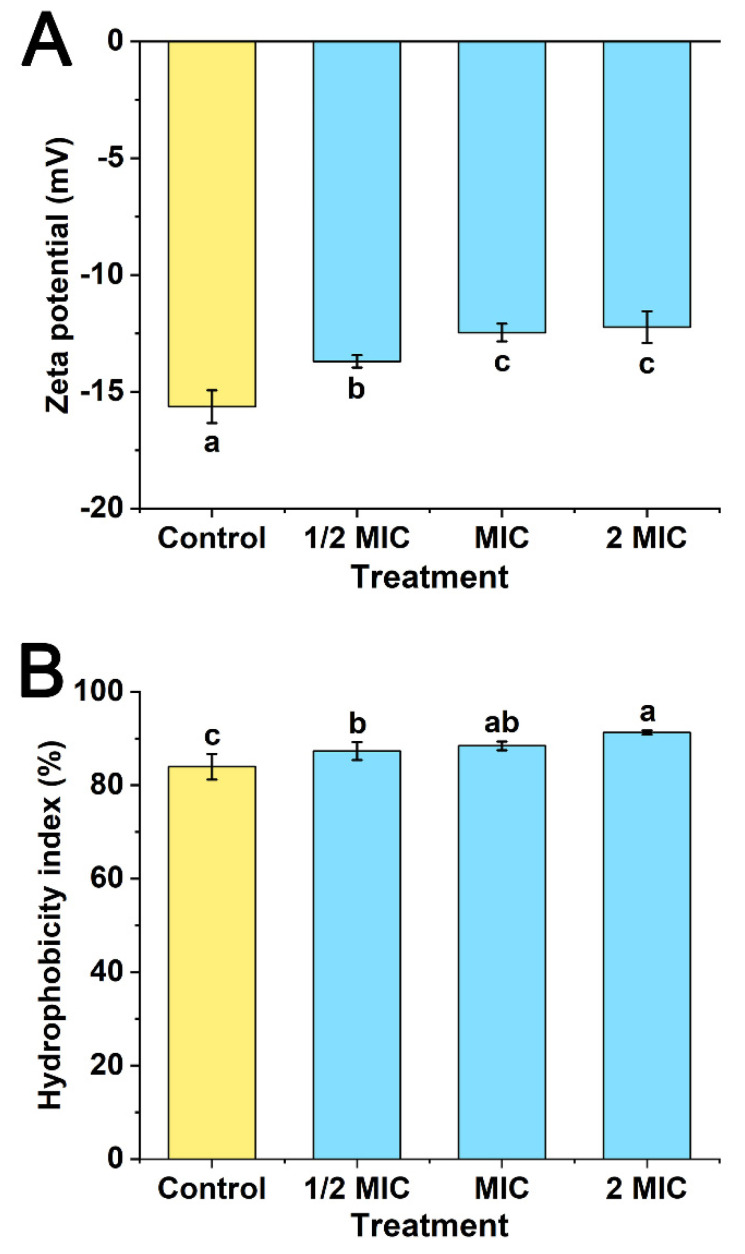
Effects of *Polygonum orientale* essential oil (concentration: 1/2MIC, MIC, and 2MIC) on zeta potential (**A**) and hydrophobicity (**B**). The group treated with DMSO (2%) served as the control. A significant difference was expressed (*p* < 0.05).

As seen in Figure 7A, the values of zeta potential increased significantly (*p* < 0.05) in the three experimental groups (1/2MIC, MIC and 2MIC) compared with the control. At MIC and 2MIC concentrations, the values of zeta potential were significantly higher (*p* < 0.05) than the 1/2MIC group. The results for the MIC and 2MIC groups revealed no significant differences.

The hydrophobicity indices of Pcc treated with POEO are shown in Figure 7B. In comparison to the control, these hydrophobicity indices increased significantly (*p* < 0.05). The hydrophobicity index of 2MIC was significantly greater (*p* < 0.05) than that of 1/2MIC. The results for the MIC and 2MIC groups revealed no significant differences.

### 3.7. Effect of POEO on Cell Wall

**Figure 8:**
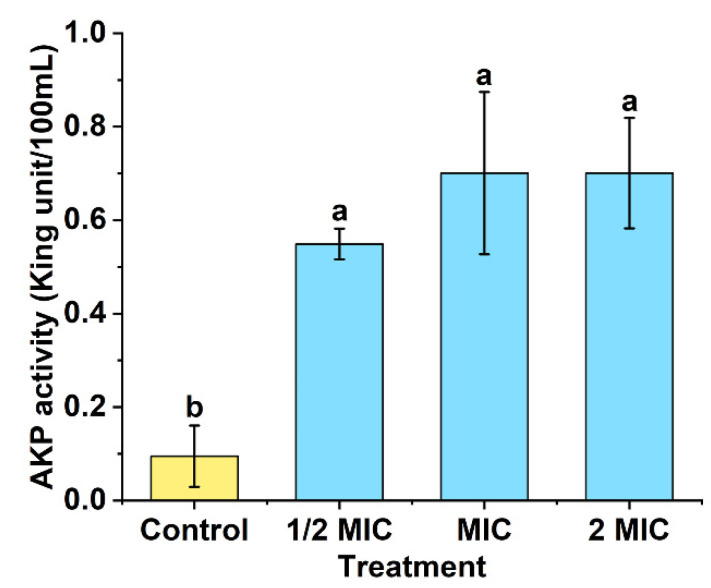
Changes of extracellular alkaline phosphatase (AKP) activity of *Pectobacterium carotovorum* subsp. *carotovorum* after treatment with *Polygonum orientale* essential oil (concentration: 1/2MIC, MIC, and 2MIC). The group treated with DMSO (2%) served as the control. A significant difference was expressed (*p* < 0.05).

The effects of POEO on cell walls were assessed by measuring extracellular AKP activity. The changes of AKP activity are shown in Figure 8. In comparison to the control group, the AKP activities of Pcc suspensions increased significantly (*p* < 0.05) in the three experimental groups (1/2MIC, MIC and 2MIC). The results for the three experimental groups showed no significant differences.

### 3.8. Effects of POEO on Cell Membrane

**Figure 9:**
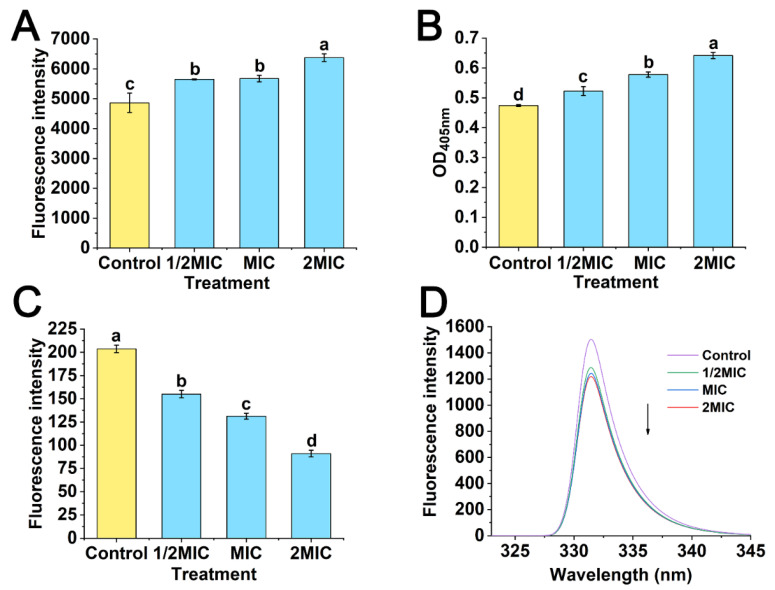
The effects of *Polygonum orientale* essential oil (concentration: 1/2MIC, MIC, and 2MIC) on cell membrane integrity (**A**), cell membrane permeability (**B**), membrane potential (**C**) and fluorescence spectra of membrane proteins (**D**) of *Pectobacterium carotovorum* subsp. *carotovorum* (Pcc). The control was Pcc treated with 2% DMSO. A significant difference was expressed (*p* < 0.05).

According to the PI staining method, the fluorescence intensity of samples can indicate cell membrane integrity. The results for cell membrane integrity are presented in Figure 9A. The fluorescence intensity of Pcc suspensions increased significantly (*p* < 0.05) in the three experimental groups (1/2MIC, MIC and 2MIC), particularly in group of 2MIC where it maximized to 6374.33.

According to the *β*-galactosidase method, OD_405_ values can indicate the permeability of the cell membrane. In Figure 9B, compared to the control, the OD_405_ values in the three experimental groups (1/2MIC, MIC and 2MIC) were significantly increased (*p* < 0.05) in an obvious gradient.

In the rhodamine fluorescence method, the fluorescence intensity of samples can indicate changes of membrane potential. Compared with the control group, the results of fluorescence intensity in the experimental groups (1/2MIC, MIC and 2MIC) declined significantly (*p* < 0.05) in an obvious gradient (Figure 9C).

The fluorescence spectra of membrane proteins are presented in Figure 9D. In the control, the maximum fluorescence intensity was 1509. Fluorescence intensity gradually declined with the increase of POEO concentrations, and the maximum values of three groups (1/2MIC, MIC and 2MIC) reduced to 1293, 1248 and 1223 respectively.

### 3.9. Effects of POEO on PK, SDH, ATPase Activities

**Figure 10:**
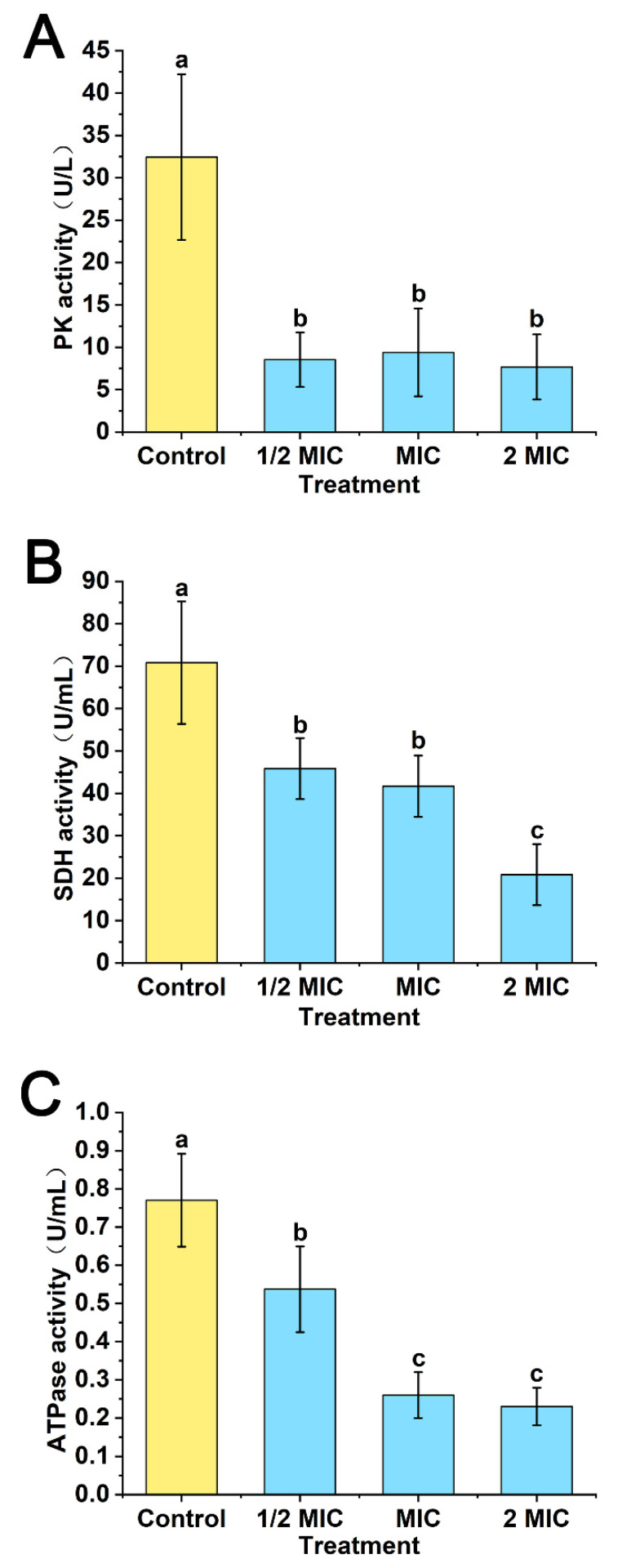
Effects of *Polygonum orientale* essential oil (concentration: 1/2MIC, MIC, and 2MIC) on pyruvate kinase (PK) activity (**A**), succinic acid dehydrogenase (SDH) activity (**B**) and adenosine triphosphatase (ATPase) activity (**C**). The group treated with DMSO (2%) served as the control. A significant difference was expressed (*p* < 0.05).

As shown in Figure 10A, the PK activities in three experimental groups (1/2MIC, MIC and 2MIC) significantly reduced (*p* < 0.05) in comparison to the control. The PK activities in the three experimental groups showed no significant differences.

As shown in Figure 10B, the SDH activities in the three experimental groups (1/2MIC, MIC and 2MIC) significantly decreased (*p* < 0.05) in comparison to the control. The SDH activity in the 2MIC group was significantly lower (*p* < 0.05) than the 1/2MIC and MIC groups.

In comparison to the control, the ATPase activities in three experimental groups (1/2MIC, MIC and 2MIC) significantly declined (*p* < 0.05) (Figure 10C). Treated with MIC and 2MIC, the ATPase activities were significantly lower (*p* < 0.05) than the 1/2MIC group. The results for the MIC and 2MIC groups showed no significant differences.

## 4. Discussion

Infected by Pcc, the quality of Chinese cabbage can severely decline. The use of chemical bactericides to control Pcc can lead to pesticide residues, thereby causing food safety problems [7]. In order to solve these problems, the search for a natural and safe antibacterial agent to control Pcc has attached much attention. EOs extracted from aromatic plants are becoming a promising kind of antimicrobial substance, because they exhibit low residues and low toxicity. In this research, the optimum extraction conditions for POEO were investigated in order to improve its extract quality. Through antibacterial activity assays, it was found that POEO had an inhibitory effect against Pcc in vitro and in vivo. Through GC-MS analysis, its chemical compounds were identified. The antibacterial mechanism of POEO was investigated, to offer a theoretical basis for the potential application of POEO in the control of Pcc.

The optimum extraction conditions of POEO were confirmed by single-factor experiments and RSM. In single-factor experiments, the effects of three factors on the extraction yield were investigated (Table 1). The relationship between soaking time and extraction yield is presented in Figure 2A. Adequate soaking was beneficial to solvent diffusion into plant cellular structures [31]. However, some essential oil volatilized when the soaking time exceeded 3 h, resulting in decrease of extraction yield (Figure 2A). The relationship between extraction time and extraction yield is presented in Figure 2B. The yield increased because the essential oil had more time to release into the extracting solution [32]. Meanwhile, the extraction temperature also continued to rise. Under higher temperature conditions, essential oil gradually degraded [33]. Thus, the yield showed a decreasing trend when extraction time exceeded 9 h (Figure 2B). The relationship between liquid to solid ratio and extraction yield is presented in Figure 2C. When the ratio of liquid to solid gradually increased, the mass transfer resistance declined and plant particles contacted better with distilled water [34]. This led to improved POEO extraction yield. After the liquid to solid ratio exceeded 10 mL/g, the heat could be used to heat up a mass of distilled water. Therefore, the extraction yield declined (Figure 2C). RSM has been widely applied to optimize extraction processes by using the multivariate quadratic regression method [35]. RSM has certain advantages, including saving time, saving reagent, reducing the number of experimental trails, and providing factor interactions [36,37]. RSM is widely applied in industry to optimize process design parameters [38]. For example, RSM has been used to optimize the extraction conditions of holy basil essential oil, *Artemisia annua* L. essential oil, and eucalyptus essential oil [39,40,41]. Because the Box-Behnken design has lower experimental costs and effective experimental design, it is superior to other RSM designs [42]. Therefore, RSM using Box-Behnken design was chosen to investigate the interactive effects of the three factors and confirm the optimum POEO extraction conditions. Through the coefficients of terms (Equation (5)), it was concluded that the order of impact of each factor was first extraction time, followed by liquid to solid ratio, and then soaking time. According to RSM, the optimal extraction conditions were 2.6 h soaking time, 7.7 h extraction time and a liquid to solid ratio of 10.3 mL/g. These conditions may be utilized during practical application.

Through the antibacterial activity assays, it was found that POEO had obvious inhibitory activity against Pcc. According to the in vitro antibacterial assay, the MIC value of POEO was 0.625 mg/mL (Table 4). Previous research indicates that certain essential oils have also shown antibacterial activity against Pcc. Hajian-Maleki et al. [43] found that six EOs had inhibitory activity against Pcc with MIC values of 15~40 mg/mL. Ashmawy et al. [44] found that the *Citharexylum spinosum* leaf extract had antibacterial activity against Pcc, and its MIC value was 1 mg/mL. The current results show that POEO has a stronger inhibitory effect against Pcc. According to in vivo antibacterial assay, POEO demonstrated effectively protective and curative activity against Pcc, compared with the control (Figure 4 and Figure 5). Almost no decay developed on Chinese cabbage stems treated with 20 mg/mL of POEO in the protective and curative assays. These results indicate the potential value of POEO for controlling Pcc.

Through GC-MS analysis, 29 different compounds were obtained and 23 compounds were identified (Table 5). The principal compounds of POEO were phytol, phytone, *n*-pentacosane, 1-octen-3-ol and *β*-ionone. As reported, the activity of EOs depended on their chemical compounds [45]. Among these compounds, phytol was more abundant than other compounds in POEO. As reported, phytol had antibacterial activities against some bacteria, including *Pseudomonas aeruginosa*, *Escherichia coli* and *Bacillus licheniformis* PKBMS_16_ [46,47]. Phytone, *n*-pentacosane, 1-octen-3-ol and *β*-ionone were also found in some EOs with antibacterial activity [48,49,50,51]. It was reported that palmitic acid had in vitro and in vivo efficacy against some bacteria, including *Klebsiella pneumoniae*, *Escherichia coli* and so on [52]. Kim et al. [53] and Kim et al. [54] found that linoleic acid had an inhibitory effect against pathogens such as *Staphylococcus aureus*, *Candida albicans* and *Pseudomonas aeruginosa*, etc. The above results of in vitro antibacterial activity assays (Table 4) and in vivo antibacterial activity assays (Figure 4 and Figure 5) prove that POEO had effective inhibitory activity against Pcc. The antibacterial activity of POEO against Pcc could depend on these compounds (phytol, phytone, *n*- pentacosane, 1-octen-3-ol, *β*-ionone, palmitic acid and linoleic acid, etc.).

The mechanism was investigated from five aspects, including cell microscopic morphology, cell surface, cell wall, cell membrane and three key enzymes in the respiratory metabolism.

Microscopic morphology of Pcc cells treated with POEO was observed by TEM. The Pcc cells treated with POEO showed the phenomena of plasmolysis, vacuoles, distortion and cell fragments (Figure 6). As lipophilic substances, EOs can easily penetrate into cell membranes and cell walls [55]. Interactions of EOs with polysaccharides, fatty acids and phospholipids make the cell membrane more permeable, resulting in the leakage of cellular contents [55]. The loss of cellular contents can increase the concentration of external solute, causing the efflux of water [56]. This may lead to changes of Pcc cell shape, such as plasmolysis (Figure 6). The leakage of cellular contents can also cause the appearance of vacuoles in Pcc (Figure 6).

Surface charge and hydrophobicity were used to determine the surface characteristics. Zeta potential reflects the stability of colloidal dispersion [57]; surface charge of bacteria was estimated by zeta potential [58]. As seen in Figure 7A, the zeta potential increased, thereby causing the cell surface to become unstable [59]. As previously reported, the adhesive ability was associated with hydrophobicity [60]. After exposure to different concentrations of POEO, the hydrophobicity indices were significantly increased (Figure 7B), indicating an increase in the adhesive ability of Pcc. Pcc cells were therefore more likely to agglutinate, thereby leading to their death.

The damage caused by POEO on cell walls was determined by measuring extracellular AKP activity. AKP is situated between the cell membrane and the cell wall. Under normal circumstances, AKP cannot pass through the integral cell wall. However, as the cell wall is damaged, AKP activity can be detected extracellularly [61]. Figure 8 reveals that the content of extracellular AKP increased, proving that the Pcc cell wall was damaged by POEO.

The integrity and permeability of cell membranes, the membrane potential and membrane protein conformation were determined, to study the action of POEO on cell membrane. The cell membrane integrity was studied by PI fluorescence staining [62]. When the cell membrane is damaged, PI can bind to nucleic acids by passing through the damaged cell membrane, thereby enhancing fluorescence [63]. The results (Figure 9A) indicated that a mass of PI penetrated cells, leading to an increase of fluorescence intensity. Therefore, cell membrane integrity was destroyed by POEO. As reported by Wichelecki et al. [64], *β*-galactosidase is a hydrolase, responsible for catalyzing the hydrolysis in microorganisms of lactose to galactose and glucose. ONPG, a lactose analogue, can pass through cell membranes and be hydrolyzed to o-nitrophenol (OPN) and galactose under the catalysis of *β*-galactosidase. OPN at 405 nm has a characteristic absorption peak [28]. The OD_405nm_ values of OPN were used to confirm the degree of cell membrane permeability. Treated with POEO, the OD_405nm_ values significantly increased (Figure 9B). The results indicated that there was considerable OPN inside the Pcc cells. Thus, ONPG was seen to penetrate the cell membrane, generating OPN in the Pcc cells under the action of *β*-galactosidase. This suggests that when treated with POEO, the permeability of cell membrane increased. MP was determined by rhodamine fluorescence. As a cationic fluorescent dye, rhodamine 123 can be used to indicate changes of MP [65]. A decrease in MP indicates the depolarization of cell membrane [66]. In this study, the fluorescence intensity obviously reduced in samples treated with POEO (Figure 9C), which indicated that the cell membrane underwent depolarization. The effect of POEO on membrane protein conformation was also investigated. Phenylalanine, tryptophan and tyrosine can emit fluorescence [67]; when membrane proteins are treated with drugs, the residues of membrane proteins are exposed, leading to a quenching effect on fluorescence [29]. Treated with POEO, the fluorescence intensity decreased (Figure 9D), which indicated that the membrane proteins of Pcc interacted with POEO and the membrane protein conformation was changed.

Respiration is a vital form of metabolism in organisms, which can convert organics into energy. The activities of three key enzymes in the respiratory metabolism were determined. As a key enzyme in the glycolytic pathway, PK catalyzes the conversion of phosphoenolpyruvate and ADP into pyruvate and ATP [68]. In the tricarboxylic acid (TCA) cycle, succinic acid can be oxidated to fumarate under the catalysis of SDH [69]. In addition, ATPase is essential for organisms to transfer substances and energy, and maintain their life activities [70]. According to the results (Figure 10), the activities of PK, SDH and ATPase were inhibited by POEO. When the activity of PK was inhibited, the conversion of phosphoenolpyruvate may have been interrupted. The inhibition of SDH might lead to the decrease of fumaric acid, thereby restraining the TCA cycle. When the ATPase was inhibited, the transfer of substances may have been restrained. As three key enzymes in respiration, the inhibition of their activity might eventually lead to reduced generation of ATP.

## 5. Conclusions

In conclusion, the optimum POEO extraction conditions (soaking time of 2.6 h, extraction time of 7.7 h and liquid to solid ratio of 10.3 mL/g) were optimized by single-factor experiments and response surface methodology. in vitro and in vivo antibacterial activity assays suggested that POEO showed effective antibacterial activity against Pcc. The result of GC-MS revealed that the major compounds of POEO were phytol (23.87%), phytone (17.24%), *n*-pentacosane (12.62%), 1-octen-3-ol (6.75%) and *β*-ionone (6.66%). Based on the results of the antibacterial mechanism, we found that POEO exerted inhibitory activity against Pcc in the following ways. Firstly, POEO can change the morphology of Pcc, causing the appearing of plasmolysis, vacuoles, cell fragments, etc. Secondly, POEO can destroy the stability of Pcc cells by increasing zeta potential and hydrophobicity. Thirdly, POEO can act on cell walls and cell membranes by causing the leakage of AKP, damaging cell membrane integrity and permeability, reducing MP, and changing membrane protein conformation. In addition, POEO could affect the respiration of Pcc by inhibiting the activities of three key enzymes (PI, SDH and ATPase) related to the TCA cycle and glycolytic pathway. Accordingly, POEO could be used to control Pcc, thereby improving the quality of Chinese cabbage.

## Figures and Tables

**Table 1:** Three factors and three levels of response surface methodology.

Factor	Levels		
Soaking time (h) *X_1_*	0(−1)	3(0)	6(1)
Extraction time (h) *X_2_*	3(−1)	6(0)	9(1)
Ratio of liquid to solid (mL/g) *X_3_*	8(−1)	10(0)	12(1)

(−1), (0) and (1) were coded levels.

**Table 2:** Box-Behnken design (BBD) for *Polygonum orientale* essential oil extraction.

Experimental Number	Factors			Extraction Yield (%) *Y*
	Soaking Time (h) *X_1_*	Extraction Time (h) *X_2_*	Ratio of Liquid to Solid (mL/g) *X_3_*	
1	0(−1)	3(−1)	10(0)	0.270
2	6(1)	3(−1)	10(0)	0.230
3	0(−1)	9(1)	10(0)	0.384
4	6(1)	9(1)	10(0)	0.367
5	0(−1)	6(0)	8(−1)	0.298
6	6(1)	6(0)	8(−1)	0.260
7	0(−1)	6(0)	12(1)	0.338
8	6(1)	6(0)	12(1)	0.318
9	3(0)	3(−1)	8(−1)	0.237
10	3(0)	9(1)	8(−1)	0.344
11	3(0)	3(−1)	12(1)	0.246
12	3(0)	9(1)	12(1)	0.358
13	3(0)	6(0)	10(0)	0.392
14	3(0)	6(0)	10(0)	0.398
15	3(0)	6(0)	10(0)	0.429
16	3(0)	6(0)	10(0)	0.399
17	3(0)	6(0)	10(0)	0.418

(−1), (0) and (1) were coded levels.

**Table 3:** Results of the analysis of variance (ANOVA).

Source	Sum of Square	Degree of Freedom	Mean Square	*F*-Value	*p*-Value
Model	0.069	9	7.70 × 10^−3^	29.86	<0.0001
*X_1_*	1.65 × 10^−3^	1	1.65 × 10^−3^	6.41	0.0392
*X_2_*	0.028	1	0.028	107.02	<0.0001
*X_3_*	1.83 × 10^−3^	1	1.83 × 10^−3^	7.09	0.0323
*X_1_X_2_*	1.32 × 10^−4^	1	1.32 × 10^−4^	0.51	0.4972
*X_1_X_3_*	8.10 × 10^−5^	1	8.10 × 10^−5^	0.31	0.5927
*X_2_X_3_*	6.25 × 10^−6^	1	6.25 × 10^−6^	0.024	0.8807
*X_1_^2^*	8.00 × 10^−3^	1	8.00 × 10^−3^	31.02	0.0008
*X_2_^2^*	0.011	1	0.011	42.2	0.0003
*X_3_^2^*	0.015	1	0.015	58.95	0.0001
Residual	1.81 × 10^−3^	7	2.58 × 10^−4^		
Lack of fit	8.31 × 10^−4^	3	2.77 × 10^−4^	1.14	0.4349
Pure error	9.75 × 10^−4^	4	2.44 × 10^−4^		
Cor Total	0.071	16			
R-Squared	97.46%				
Adj R-Squared	94.20%				

Symbols *X_1_*, *X_2_* and *X_3_* represented as soaking time, extraction time and liquid to solid ratio. *p* < 0.01: highly significant; 0.01 < *p* < 0.05: significant; *p* > 0.05: not significant.

**Table 4:** Minimum inhibitory concentration (MIC) of *Polygonum orientale* essential oil against *Pectobacterium carotovorum* subsp. *carotovorum* (Pcc).

Strain	Concentration (mg/mL)													3% DMSO
	40	20	10	5	2.5	1.25	0.625	0.313	0.156	0.08	0.04	0.02	0.01	
Pcc	-	-	-	-	-	-	-	+	+	+ +	+ + +	+ + +	+ + +	+ + +

3% DMSO served as a control. “-” represents no growth of Pcc; “+” represents weak growth of Pcc; “+ +” represents moderate growth of Pcc; + + +, represents extreme growth of Pcc.

**Table 5:** Chemical compositions of *Polygonum orientale* essential oil.

Peak	Compound	RT (min)	RI_1_	RI_2_	Molecular Formula	Relative Percentage (%)
1	1-octen-3-ol	4.18	961.1	980 ± 2	C_8_H_16_O	6.75
2	unidentified	5.08	1014.6	-	-	1.79
3	3,5-octadien-2-one	6.38	1092.5	1091 ± 10	C_8_H_12_O	0.49
4	unidentified	7.99	1159.0	-	-	0.58
5	safranal	9.17	1205.0	1201 ± 4	C_10_H_14_O	0.63
6	2-methyl-2-decen-4-one	9.48	1215.9	1215 ± N/A	C_10_H_18_O	0.36
7	*β*-cyclocitral	9.74	1225.3	1220 ± 3	C_10_H_16_O	1.05
8	*β*-homocyclocitral	10.78	1261.8	1254 ± 3	C_11_H_18_O	0.47
9	geranyl acetone	16.45	1454.0	1456 ± 5	C_13_H_22_O	2.21
10	*β*-ionone	17.51	1490.1	1491 ± 2	C_13_H_20_O	6.66
11	unidentified	21.48	1628.9	-	-	0.69
12	unidentified	22.08	1651.0	-	-	0.86
13	*n*-heptadecane	23.45	1700.4	1700	C_17_H_36_	0.73
14	unidentified	25.54	1779.1	-	-	0.62
15	*n*-octadecane	26.10	1800.4	1800	C_18_H_38_	0.53
16	phytone	27.29	1847.7	1844 ± 4	C_18_H_36_O	17.24
17	diisobutyl phthalate	27.88	1871.0	1870 ± 4	C_16_H_22_O_4_	2.95
18	farnesyl acetone	29.11	1921.2	1919 ± 5	C_18_H_30_O	3.35
19	unidentified	29.27	1927.8	-	-	1.12
20	isophytol	29.80	1950.5	1948 ± 2	C_20_H_40_O	2.09
21	palmitic acid	30.26	1969.5	1968 ± 7	C_16_H_32_O_2_	3.21
22	*n*-heneicosane	33.36	2100.5	2100	C_21_H_44_	3.01
23	phytol	33.72	2116.8	2116 ± 2	C_20_H_40_O	23.87
24	linoleic acid	34.56	2154.8	2133 ± 12	C_18_H_32_O_2_	0.43
25	*n*-tricosane	37.70	2308.1	2300	C_23_H_48_	1.53
26	*n*-tetracosane	39.73	2404.3	2400	C_24_H_50_	0.67
27	*n*-pentacosane	41.73	2502.7	2500	C_25_H_52_	12.62
28	*n*-hexacosane	43.60	2607.6	2600	C_26_H_54_	0.78
29	*n*-heptacosane	45.43	2700.7	2700	C_27_H_56_	2.71

RT: Retention time; RI_1_: Calculated retention indices; RI_2_: Retention indices in NIST MS Search 2.2 database.

## Data Availability

Data is contained within the article or Supplementary Material.

## Supplementary Materials

Supporting information can be downloaded at: www.mdpi.com/article/10.3390/foods11111585/s1, Table S1: The meanings of acronyms appearing in this article.

## References

[1] Managa M.G., Remize F., Garcia C., Sivakumar D. (2019). Effect of moist cooking blanching on colour, phenolic metabolites and glucosinolate content in Chinese cabbage (*Brassica rapa* L. subsp. *chinensis*). Foods.

[2] Lim J.A., Jee S., Lee D.H., Roh E., Jung K., Oh C., Heu S. (2013). Biocontrol of *Pectobacterium carotovorum* subsp. *carotovorum* using bacteriophage PP1. J. Microbiol. Biotechnol..

[3] Liu M.Y., Wu F., Wang S., Lu Y., Chen X.P., Wang Y.H., Gu A.X., Zhao J.J., Shen S.X. (2019). Comparative transcriptome analysis reveals defense responses against soft rot in Chinese cabbage. Hortic. Res..

[4] Tsuda K., Tsuji G., Higashiyama M., Ogiyama H., Umemura K., Mitomi M., Kubo Y., Kosaka Y. (2016). Biological control of bacterial soft rot in Chinese cabbage by *Lactobacillus plantarum* strain BY under field conditions. Biol. Control.

[5] Dai Y.N., Liu Q.H., Pan H., Zhang Y.F., Pu J.F., Bai J.P. (2020). Analysis on the control effect of registered pesticides of soft rot of Chinese cabbage. Agric. Technol..

[6] Guo Z.H., Wang Q.X. (2017). Efficacy of ozonated water against *Erwinia carotovora* subsp. *carotovora* in *Brassica campestris* ssp. *chinensis*. Ozone-Sci. Eng..

[7] Han X.B., Zhao J., Cao J.M., Zhang C.S. (2019). Essential oil of *Chrysanthemum indicum* L.: Potential biocontrol agent against plant pathogen *Phytophthora nicotianae*. Environ. Sci. Pollut. Res..

[8] Dhifi W., Bellili S., Jazi S., Bahloul N., Mnif W. (2016). Essential oils’ chemical characterization and investigation of some biological activities: A critical review. Medicines.

[9] Benomari F.Z., Andreu V., Kotarba J., El Amine Dib M., Bertrand C., Muselli A., Costa J., Djabou N. (2017). Essential oils from Algerian species of *Mentha* as new bio-control agents against phytopathogen strains. Environ. Sci. Pollut. R..

[10] Ju J., Xie Y.F., Yu H., Guo Y.H., Cheng Y.L., Qian H., Yao W.R. (2020). Synergistic interactions of plant essential oils with antimicrobial agents: A new antimicrobial therapy. Crit. Rev. Food Sci..

[11] Zhang L.L., Zhang L.F., Hu Q.P., Hao D.L., Xu J.G. (2017). Chemical composition, antibacterial activity of *Cyperus rotundus* rhizomes essential oil against *Staphylococcus aureus* via membrane disruption and apoptosis pathway. Food Control.

[12] Morshdy A.E.M.A., Al-Mogbel M.S., Mohamed M.E.M., Elabbasy M.T., Elshafee A.K., Hussein M.A. (2021). Bioactivity of essential oils for mitigation of *Listeria monocytogenes* isolated from fresh retail chicken meat. Foods.

[13] Wajs-Bonikowska A., Malarz J., Szoka L., Kwiatkowski P., Stojakowska A. (2021). Composition of essential oils from roots and aerial parts of *Carpesium cernuum* and their antibacterial and cytotoxic activities. Molecules.

[14] Hu G.F., Liu M.Y., Shen H.M., Zhang X.R., Li Y.Q., Niu S.J. (2011). A study on contact toxicity of extracts from *Polygonum orientale* against 13 species of agricultural pests. Acta Prataculturae Sin..

[15] Malik U., Barik A. (2015). Free fatty acids from the weed, *Polygonum orientale* leaves for attraction of the potential biocontrol agent, *Galerucella placida* (Coleoptera: Chrysomelidae). Biocontrol Sci. Technol..

[16] Liu Z.Z., Li H.L., Zhu Z., Huang D., Qi Y.L., Ma C.H., Zou Z.R., Ni H.Y. (2022). *Cinnamomum camphora* fruit peel as a source of essential oil extracted using the solvent-free microwave-assisted method compared with conventional hydrodistillation. Lwt-Food Sci. Technol..

[17] Haddouchi F., Chaouche T.M., Zaouali Y., Ksouri R., Attou A., Benmansour A. (2013). Chemical composition and antimicrobial activity of the essential oils from four *Ruta* species growing in Algeria. Food Chem..

[18] Zhang Y.B., Liu X.Y., Wang Y.F., Jiang P.P., Quek S.Y. (2016). Antibacterial activity and mechanism of cinnamon essential oil against *Escherichia coli* and *Staphylococcus aureus*. Food Control.

[19] Ambrico A., Trupo M., Magarelli R., Balducchi R., Ferraro A., Hristoforou E., Marino T., Musmarra D., Casella P., Molino A. (2020). Effectiveness of *Dunaliella salina* extracts against *Bacillus subtilis* and bacterial plant pathogens. Pathogens.

[20] Zhang Z.Y., Dai G.H., Zhuge Y.Y., Li Y.B. (2008). Protective effect of *Robinia pseudoacacia* Linnl extracts against cucumber powdery mildew fungus, *Sphaerotheca fuliginea*. Crop. Prot..

[21] Cui W.Y., He P.J., Munir S., He P.B., He Y.Q., Li X.Y., Yang L.J., Wang B., Wu Y.X., He P.F. (2019). Biocontrol of soft rot of Chinese cabbage using an endophytic bacterial strain. Front. Microbiol..

[22] Li H.Y., Luo Y., Zhang X.S., Shi W.L., Gong Z.T., Shi M., Chen L.L., Chen X.L., Zhang Y.Z., Song X.Y. (2014). Trichokonins from *Trichoderma pseudokoningii* SMF2 induce resistance against gram-negative *Pectobacterium carotovorum* subsp. *carotovorum* in Chinese cabbage. Fems Microbiol. Left.

[23] Bajer T., Šilha D., Ventura K., Bajerová P. (2017). Composition and antimicrobial activity of the essential oil, distilled aromatic water and herbal infusion from *Epilobium parviflorum* Schreb. Ind. Crops Prod..

[24] Sun X.H., Hao L.R., Xie Q.C., Lan W.Q., Zhao Y., Pan Y.J., Wu V.C.H. (2020). Antimicrobial effects and membrane damage mechanism of blueberry (*Vaccinium corymbosum* L.) extract against *Vibrio parahaemolyticus*. Food Control.

[25] Huang H., Wang D., Belwal T., Dong L., Lu L., Zou Y., Li L., Xu Y.Q., Luo Z.S. (2021). A novel W/O/W double emulsion co-delivering brassinolide and cinnamon essential oil delayed the senescence of broccoli via regulating chlorophyll degradation and energy metabolism. Food Chem..

[26] Zhao J.X., Peng T., Liang S.B., Ma M.M., Zeng Z.L., Yu P., Gong D.M., Deng S.G. (2020). Antibacterial activity and action mechanism of microencapsulated dodecyl gallate with methyl-*β*-cyclodextrin. Food Control.

[27] Kong J., Zhang Y., Ju J., Xie Y.F., Guo Y.H., Cheng Y.L., Qian H., Quek S.Y., Yao W.R. (2019). Antifungal effects of thymol and salicylic acid on cell membrane and mitochondria of *Rhizopus stolonifer* and their application in postharvest preservation of tomatoes. Food Chem..

[28] Cui H.Y., Bai M., Sun Y.H., Abdel-Samie M.A.S., Lin L. (2018). Antibacterial activity and mechanism of Chuzhou chrysanthemum essential oil. J. Funct. Foods.

[29] Li L., Song X., Yin Z.Q., Jia R.Y., Li Z.W., Zhou X., Zou Y.F., Li L.X., Yin L.Z., Yue G.Z. (2016). The antibacterial activity and action mechanism of emodin from *Polygonum cuspidatum* against *Haemophilus parasuis* in vitro. Microbiol. Res..

[30] Guo F.Y., Chen Q.P., Liang Q., Zhang M., Chen W.X., Chen H.M., Yun Y.H., Zhong Q.P., Chen W.J. (2021). Antimicrobial activity and proposed action mechanism of linalool against *Pseudomonas fluorescens*. Front. Microbiol..

[31] Ji S., Wang Y.J., Gao S.K., Shao X., Cui W., Du Y., Guo M.Z., Tang D.Q. (2019). Highly efficient and selective extraction of minor bioactive natural products using pure ionic liquids: Application to prenylated flavonoids in licorice. J. Ind. Eng. Chem..

[32] Madhumita M., Guha P., Nag A. (2019). Optimization of the exhaustive hydrodistillation method in the recovery of essential oil from fresh and cured betel leaves (*Piper betle* L.) using the Box-Behnken design. J. Food Process. Preserv..

[33] Wang F.Y., You H.Q., Guo Y.H., Wei Y.K., Xia P.G., Yang Z.Q., Ren M., Guo H., Han R.L., Yang D.F. (2020). Essential oils from three kinds of fingered citrons and their antibacterial activities. Ind. Crops Prod..

[34] Milojević S.Ž., Stojanović T.D., Palić R., Lazić M.L., Veljković V.B. (2008). Kinetics of distillation of essential oil from comminuted ripe juniper (*Juniperus communis* L.) berries. Biochem. Eng. J..

[35] Yu H., Wang C., Deng S.T., Bi Y.G. (2017). Optimization of ultrasonic-assisted extraction and UPLC-TOF/MS analysis of limonoids from lemon seed. LWT-Food Sci. Technol..

[36] Akalin M.K., Tekin K., Akyüz M., Karagöz S. (2015). Sage oil extraction and optimization by response surface methodology. Ind. Crops Prod..

[37] Wang H.W., Liu Y.Q., Wei S.L., Yan Z.J. (2012). Application of response surface methodology to optimise supercritical carbon dioxide extraction of essential oil from *Cyperus rotundus* Linn. Food Chem..

[38] Hamid H.A., Jenidi Y., Thielemans W., Somerfield C., Gomes R.L. (2016). Predicting the capability of carboxylated cellulose nanowhiskers for the remediation of copper from water using response surface methodology (RSM) and artificial neural network (ANN) models. Ind. Crops Prod..

[39] Sutaphanit P., Chitprasert P. (2014). Optimisation of microencapsulation of holy basil essential oil in gelatin by response surface methodology. Food Chem..

[40] Li Y.T., Xia L., Vazquez J.F.T., Song S.X. (2017). Optimization of supercritical CO_2_ extraction of essential oil from *Artemisia annua* L. by means of response surface methodology. J. Essent. Oil Bear. Plants.

[41] Ning X., Yue S.L. (2019). Optimization of preparation conditions of eucalyptus essential oil microcapsules by response surface methodology. J. Food Process. Preserv..

[42] Pongsumpun P., Iwamoto S., Siripatrawan U. (2019). Response surface methodology for optimization of cinnamon essential oil nanoemulsion with improved stability and antifungal activity. Ultrason. Sonochem..

[43] Hajian-Maleki H., Baghaee-Ravari S., Moghaddam M. (2021). Herbal essential oils exert a preservative effect against the potato soft rot disease. Sci. Hortic..

[44] Ashmawy N.A., Salem M.Z.M., EL-Hefny M., Abd El-Kareem M.S.M., El-Shanhorey N.A., Mohamed A.A., Salem A.Z.M. (2018). Antibacterial activity of the bioactive compounds identified in three woody plants against some pathogenic bacteria. Microb. Pathogenesis..

[45] Bellik F.Z., Benkaci-Ali F., Alsafra Z., Eppe G., Tata S., Sabaou N., Zidani R. (2019). Chemical composition, kinetic study and antimicrobial activity of essential oils from *Cymbopogon schoenanthus* L. *Spreng* extracted by conventional and microwave-assisted techniques using cryogenic grinding. Ind. Crops Prod..

[46] Islam M.T., Ali E.S., Uddin S.J., Shaw S., Islam M.A., Ahmed M.I., Shill M.C., Karmakar U.K., Yarla N.S., Khan I.N. (2018). Phytol: A review of biomedical activities. Food Chem. Toxicol..

[47] Saha M., Bandyopadhyay P.K. (2020). in vivo and in vitro antimicrobial activity of phytol, a diterpene molecule, isolated and characterized from *Adhatoda vasica* Nees. (Acanthaceae), to control severe bacterial disease of ornamental fish, *Carassius auratus*, caused by *Bacillus licheniformis* PKBMS_16_. Microb. Pathog..

[48] Al-Rowaily S.L., Abd-ElGawad A.M., Assaeed A.M., Elgamal A.M., El Gendy A.G., Mohamed T.A., Dar B.A., Mohamed T.K., Elshamy A.I. (2020). Essential oil of *Calotropis procera*: Comparative chemical profiles, antimicrobial activity, and allelopathic potential on weeds. Molecules.

[49] Hossain M.A., Ismail Z., Rahman A., Kang S.C. (2008). Chemical composition and anti-fungal properties of the essential oils and crude extracts of *Orthosiphon stamineus* Benth. Ind. Crops Prod..

[50] Li R., Hu H.B., Li X.F., Zhang P., Xu Y.K., Yang J.J., Wang Y.F. (2015). Essential oils composition and bioactivities of two species leaves used as packaging materials in Xishuangbanna, China. Food Control.

[51] Matebie W.A., Zhang W.C., Xie G.B. (2019). Chemical composition and antimicrobial activity of essential oil from *Phytolacca dodecandra* collected in Ethiopia. Molecules.

[52] Padmini N., Rashiya N., Sivakumar N., Kannan N.D., Manjuladevi R., Rajasekar P., Prabhu N.M., Selvakumar G. (2020). in vitro and in vivo efficacy of methyl oleate and palmitic acid against ESBL producing MDR *Escherichia coli* and *Klebsiella pneumoniae*. Microb. Pathog..

[53] Kim H.S., Cha E., Ham S.Y., Park J.H., Nam S., Kwon H., Byun Y., Park H.D. (2021). Linoleic acid inhibits *Pseudomonas aeruginosa* biofilm formation by activating diffusible signal factor-mediated quorum sensing. Biotechnol. Bioeng..

[54] Kim Y.G., Lee J.H., Park J.G., Lee J. (2020). Inhibition of *Candida albicans* and *Staphylococcus aureus* biofilms by centipede oil and linoleic acid. Biofouling.

[55] Raut J.S., Karuppayil S.M. (2014). A status review on the medicinal properties of essential oils. Ind. Crops Prod..

[56] Pilizota T., Shaevitz J.W. (2013). Plasmolysis and cell shape depend on solute outer-membrane permeability during hyperosmotic shock in *E. coli*. Biophys. J..

[57] Hosseini S.F., Ghaderi J., Gómez-Guillén M.C. (2022). Tailoring physico-mechanical and antimicrobial/antioxidant properties of biopolymeric films by cinnamaldehyde-loaded chitosan nanoparticles and their application in packaging of fresh rainbow trout fillets. Food Hydrocolloid..

[58] Maillard A.P.V.F., Espeche J.C., Maturana P., Cutro A.C., Hollmann A. (2021). Zeta potential beyond materials science: Applications to bacterial systems and to the development of novel antimicrobials. BBA-Biomembranes.

[59] Moo C.L., Yang S.K., Osman M.A., Yuswan M.H., Loh J.Y., Lim W.M., Lim S.H.E., Lai K.S. (2020). Antibacterial activity and mode of action of *β*-caryophyllene on *Bacillus cereus*. Pol. J. Microbiol..

[60] Tang C.L., Chen J.L., Zhang L.X., Zhang R.F., Zhang S.C., Ye S.X., Zhao Z.M., Yang D.P. (2020). Exploring the antibacterial mechanism of essential oils by membrane permeability, apoptosis and biofilm formation combination with proteomics analysis against methicillin-resistant *staphylococcus aureus*. Int. J. Med. Microbiol..

[61] Wang F., Wei F.Y., Song C.X., Jiang B., Tian S.Y., Yi J.W., Yu C.L., Song Z.B., Sun L.G., Bao Y.L. (2017). *Dodartia orientalis* L. essential oil exerts antibacterial activity by mechanisms of disrupting cell structure and resisting biofilm. Ind. Crops Prod..

[62] Wang F., Liu H., Li J.Y., Zhang W.W., Jiang B., Xuan H.Z. (2021). Australian propolis ethanol extract exerts antibacterial activity against methicillin-resistant *Staphylococcus aureus* by mechanisms of disrupting cell structure, reversing resistance, and resisting biofilm. Braz. J. Microbiol..

[63] Yan F.L., Dang Q.F., Liu C.S., Yan J.Q., Wang T., Fan B., Cha D.S., Li X.L., Liang S.N., Zhang Z.Z. (2016). 3,6-*O*-[*N*-(2-aminoethyl)-acetamide-yl]-chitosan exerts antibacterial activity by a membrane damage mechanism. Carbohyd. Polym..

[64] Wichelecki D.J., McNew T.M., Aygun A., Torrey K., Stephenson L.D. (2011). Detection of liposome lysis utilizing an enzyme–substrate system. Appl. Biochem. Biotechnol..

[65] Xu C., Li J.L., Yang L.Q., Shi F., Yang L., Ye M. (2017). Antibacterial activity and a membrane damage mechanism of *Lachnum* YM30 melanin against *Vibrio parahaemolyticus* and *Staphylococcus aureus*. Food Control.

[66] Liu X., Cai J.X., Chen H.M., Zhong Q.P., Hou Y.Q., Chen W.J., Chen W.X. (2020). Antibacterial activity and mechanism of linalool against *Pseudomonas aeruginosa*. Microb. Pathog..

[67] Kong M., Chen X.G., Liu C.S., Liu C.G., Meng X.H., Yu L.J. (2008). Antibacterial mechanism of chitosan microspheres in a solid dispersing system against *E. coli*. Colloids Surf. B.

[68] Wang Y.Y., Liu R., Hou Q., Tian X.N., Fan X.Q., Zhang W.G., Zhou G.H. (2020). Comparison of activity, expression and S-nitrosylation of glycolytic enzymes between pale, soft and exudative and red, firm and non-exudative pork during post-mortem aging. Food Chem..

[69] Ju J., Xie Y.F., Yu H., Guo Y.H., Cheng Y.L., Zhang R.R., Yao W.R. (2020). Major components in *Lilac* and *Litsea cubeba* essential oils kill *Penicillium roqueforti* through mitochondrial apoptosis pathway. Ind. Crops Prod..

[70] Bajpai V.K., Sharma A., Baek K.H. (2013). Antibacterial mode of action of *Cudrania tricuspidata* fruit essential oil, affecting membrane permeability and surface characteristics of food-borne pathogens. Food Control.

