# Supplementary material for: Antibacterial Activity and Mechanism of Polygonum orientale L. Essential Oil against Pectobacterium carotovorum subsp. carotovorum
Source: Foods. 2022 May 28;11(11):1585. doi: 10.3390/foods11111585 (PMC9180537; doi:10.3390/foods11111585)
Supplement: Supplementary file 1 [file foods-11-01585-s001.zip › foods-1737205-supplementary.pdf]

Table S1. The meanings of acronyms appearing in this article.

Acronyms	Meanings
Pcc	*Pectobacterium carotovorum* subsp. *carotovorum*
POEO	*Polygonum orientale* L. essential oil
MIC	minimum inhibitory concentration
GC-MS	gas chromatography-mass spectrometer
PK	pyruvate kinase
SDH	succinate dehydrogenase
EOs	essential oils
ATPase	adenosine triphosphatase
RSM	response surface methodology
BBD	Box-Behnken design
ANOVA	analysis of variance
TEM	transmission electron microscope
OD	optical density
AKP	alkaline phosphatase
PI	propidium iodide
MP	membrane potential
ONPG	*o*-nitrophenyl-*β*,*D*-galactopyranoside
OPN	*o*-nitrophenol
ADP	adenosine diphosphate
ATP	adenosine triphosphate
TCA	tricarboxylic acid
